# Live tutoring calls did not improve learning during the COVID-19 pandemic in Sierra Leone^☆^

**DOI:** 10.1016/j.jdeveco.2023.103114

**Published:** 2023-09

**Authors:** Lee Crawfurd, David K. Evans, Susannah Hares, Justin Sandefur

**Affiliations:** a Center for Global Development, London, UK; b Center for Global Development, Washington DC, USA

**Keywords:** Education, COVID, Distance learning, Teachers

## Abstract

Education systems regularly face unexpected school closures, whether due to disease outbreaks, natural disasters, or other adverse shocks. In low-income countries where internet access is scarce, distance learning – the most common educational solution – is often passive, via TV or radio, with little opportunity for teacher–student interaction. In this paper we evaluate the effectiveness of live tutoring calls from teachers, designed to supplement radio instruction during the 2020 school closures prompted by the COVID-19 pandemic. We do this with a randomised controlled trial with 4,399 primary school students in Sierra Leone. Tutoring calls led to some limited increase in educational activity, but had no effect on mathematics or language test scores, whether for girls or boys, and whether provided by public or private school teachers. Even having received tutoring calls, one in three children reported not listening to educational radio at all, so limited take-up may partly explain our results.

## Highlights

• During the COVID pandemic, schools in Sierra Leone closed for in-person instruction.
• Radio education was the principal substitute supplied.
• We evaluate live tutoring calls from teachers, designed to supplement radio lessons.
• Tutoring calls increased engagement in educational activity but not test scores.
• Null effects were found for girls and boys, and public and private school teachers.

## 1 Introduction

Schools closed worldwide in March 2020 in response to the COVID-19 pandemic. These closures present a number of policy challenges for governments. First, children miss out on direct learning in classrooms. In several countries, children lost ten percent or more of the total time they were expected to spend in-person at school over the course of their lives (Evans et al., 2021). Second, children may forget much of what they already learned in school. In high- and low-income countries, students – especially lower income students – regress in their academic skills during academic breaks (Alexander et al., 2007, Slade et al., 2017). Third, some may not return once schools re-open: following closures due to the 2014–2016 Ebola crisis, re-enrolment in Sierra Leone was high but imperfect (Kastelic et al., 2015), and gross enrolment fell slightly from 2013 to 2015 (World Bank, 2021).

To keep students engaged and learning during school closures, governments around the world abruptly shifted to distance learning. In low-income countries, the medium with the greatest reach is radio. The Government of Sierra Leone, like many others, announced a national radio teaching programme shortly after schools closed in March 2020. Many governments and partners complemented radio teaching with SMS-based reminders. In Sierra Leone, a number of non-state organisations, including our implementation partner Rising Academy Network, supported the Government to develop content for the radio teaching programme and provided SMS reminders. Missing from these and similar efforts, however, is any direct interaction between teachers and students. Teachers add significant value to student learning (Chetty et al., 2014), but little of that value is explained by teacher characteristics (Bau and Das, 2020), suggesting that effective interactions may drive teacher effects.

In this paper we evaluate the effectiveness of live phone tutorials in increasing engagement with radio lessons, improving learning, and ultimately ensuring that children re-enrol in school upon re-opening. We compare phone tutorials delivered by private-school teachers to those delivered by government-school teachers.

To do this we designed a randomised control trial, in which 4,399 students were randomly assigned to one of three treatment groups. The first group received SMS reminders to listen to educational radio. The second group received SMS reminders and weekly phone tutorials from private school teachers. The third group received SMS reminders and weekly phone tutorials from government school teachers. We also cross-randomise survey mode, with a sub-sample of 500 students assigned to be surveyed and tested in-person rather than by phone.

We find no effect of calls by either private or government teachers on mathematics or language test scores. This is robust to controls for student characteristics and school fixed effects, and differences in survey mode (in-person or phone). We do find some positive effects of tutoring calls on educational engagement by parents and children. Tutoring calls increase an index of student activity by 0.29 standard deviations and an index of parent activity by 0.27 standard deviations. Within this index, however, we see no statistically significant effect on the probability that children listened to educational radio, and only a 4 percentage point increase (significant at the 10 percent level) in the probability that their parent knew the correct FM frequency.

Re-enrolment was over 99 percent even in the control group, so we see no effect of calls on re-enrolment. Private school teachers (who were directly managed by the project implementer) did exert greater effort than the government school teachers. Private school teachers placed 59 percent of planned calls, compared to 41 percent for government school teachers. But this extra effort did not translate to any difference in student outcomes. This lack of a difference in outcomes across implementers – despite a difference in implementation fidelity – suggests that the null finding results not from an implementation failure but in the effectiveness of the intervention itself. Many students did not listen to educational radio at all (43 percent in the control group and 34 percent in the treatment group), so low uptake of the educational radio is potential part of the reason for a lack of impact on student learning.

Our results join several other studies that evaluate the effect of phone tutorials during the COVID-19 pandemic (as well as others in process). The first found mathematics learning gains in Botswana of between 0.16 and 0.29 standard deviations for an SMS and live phone call intervention. Students were sent mathematics problems by SMS and then called by NGO staff to work through the problems (Angrist et al., 2022). A second study replicated the Botswana findings in Nepal, with a similar intervention improving numeracy test scores by around 0.2 standard deviations (Radhakrishnan et al., 2021). The third found large effects of 0.56 standard deviations in mathematics and 0.66 standard deviations in literacy in Bangladesh. The intervention was a 30-minute telephone mentoring session with a student volunteer from a local university (Hassan et al., 2021). The fourth by contrast to the others found no benefits on mathematics performance in Kenya of either short 5-minute “accountability checks” or 15-minute tutoring calls (Schueler and Rodriguez-Segura, 2021).

Why do we see different effects between the three studies with positive effects (Angrist et al. in Botswana, Hassan et al. in Bangladesh, and Radhakrishnan et al. in Nepal) and the two that did not (Schueler and Rodriguez-Segura in Kenya, and this paper on Sierra Leone)? We discuss four possibilities.

First, the intervention that we study is focused on encouraging engagement with radio instruction, following guides to review material covered in radio broadcasts. One possibility is therefore that tying tuition calls to radio instruction is less effective than designing more personalised instruction (Banerjee et al., 2007, Banerjee et al., 2017a). Calls followed a set script. After introductions, teachers would ask children a set of questions related to the most recent radio episode. In literacy (lower primary level), these questions would test the child’s ability to hear words, syllables, and letter sounds, their ability to spell short words, and practice speaking. In mathematics (lower primary level), children were asked to practice counting and simple arithmetic operations.

Second, the programme in Sierra Leone did not generate high engagement by either parents or students, with the radio lessons or other educational activities. The programme was implemented with moderate fidelity: over 80% of parents in the treatment group recalled receiving the calls. But we found only moderate increases in an index of parental or child educational engagement, including no significant effect on engagement with the radio lessons, as well as no significant effect on overall time spent in educational activity. In Kenya, students substituted time spent away from other forms of studying to the roughly 20-minute tutorial calls. In Bangladesh a key focus of the intervention was on increasing the engagement of mothers with their child’s learning, which increased by 22 minutes per day.

Third, and perhaps relatedly, the two studies with null results used primary school teachers to deliver calls, and the two studies with positive results used NGO staff (Angrist et al., 2020b) and university students (Hassan et al., 2021) who may have been more highly skilled or had more targeted training.

Fourth, the studies that worked asked families to opt in, whereas those that did not attempted to work with all the children enrolled in the relevant schools and grades. Treatment effects could be larger for more motivated students (and families) who chose to opt-in to distance learning, than for those who would not have chosen to opt-in. This theory is not, however, supported by our heterogeneity analysis which finds little heterogeneity by parent characteristics. The rate of opt-in was also high in Botswana, with a sample that was observationally similar to the broader population.

Prior to the COVID crisis, some literature had considered other forms of low-cost distance-learning and digital communication. For example a radio-based math instruction programme in Nicaragua in the late 1970s increased test scores (Jamison et al., 1981). A radio learning programme in Sierra Leone during the Ebola epidemic helped to keep children connected to education, but the lack of adult support to children was cited as a key weakness of this programme (Barnett et al., 2018).

SMS reminder messages have proven effective at improving educational outcomes in some contexts. Nudges in Brazil reduced dropout in 2020 (Lichand and Christen, 2021). Weekly SMS messages and monthly quizzes in rural China improved student academic outcomes (Mo et al., 2014). SMS messages and phone calls can also useful for engaging parents during normal times (Barrera et al., 2020, Berlinski et al., 2021, Kraft and Rogers, 2015, Doss et al., 2018). But the size of text message effects tends to be modest relative to the impact of in-person interactions with teachers (Araujo et al., 2016, Bau and Das, 2020).

Our study also adds to the little that has been written on assessing learning by phone (Angrist et al., 2020a). We find that in Sierra Leone, phone-based assessments are feasible, that they have good internal reliability, and that using in-person versus phone assessment does not affect our estimates of treatment. Two studies in Kenya and Cote D’Ivoire compare in-person and phone-based assessments of the same individuals, finding high internal reliability of phone-based assessments but low (Rodriguez-Segura and Schueler, 2022) to medium (Sobers et al., 2021) correlation with in-person results. Another study shows the reliability of phone-based assessment through randomising different questions to test the same underlying proficiency, and using a real-effort task to disentangle cognitive skills from effort (Angrist et al., 2022). There is experimental evidence that phone surveys on other topics can be reliable (Garlick et al., 2020), and other research from developing countries showing that survey mode (e.g., paper versus computer-assisted) does make a difference for measured outcomes in both education (Singh, 2020) and other sectors (Caeyers et al., 2012). Yet phone-based assessment offers potential for significant cost-savings over in-person learning assessments (Angrist et al., 2020a). For example phone-based assessments trialled in India during the COVID-19 crisis cost USD 3.5 per student, compared with typical in-person costs of around USD 5–13 per student (Joshi and Sharma, 2021).

The rest of this paper is structured as follows; Section 2 provides more background about the programme context, Section 3 discusses the interventions, Section 4 outlines the experimental design, Section 5 the data, Section 6 the results, and Section 7 concludes.

## 2 Background

The COVID-19 crisis affected Sierra Leone much as it did many of the country’s neighbours. Sierra Leone recorded a total of 2611 confirmed cases and 76 deaths in 2020 (Dong et al., 2020). Awareness of COVID-19 was high (Fitzpatrick et al., 2020). The economic impact was severe—small business profits fell by 50 percent between March and June 2020, and average wage earnings fell 20 percent, with increases in household debt and reduced food consumption (Meriggi et al., 2020).

Turning to education, students globally lost an average of two-thirds of an academic year of schooling in 2020 (UNESCO, 2021). In some low-income countries, losing this much education can represent a substantive proportion of children’s total lifetime expected schooling (Evans et al., 2021). In Sierra Leone, schools closed on 31st March 2020 until further notice. Primary schools reopened for exam grades (Grade 6) only on 1st July 2020. Schools re-opened for all children for the next academic year on 5th October 2020. A nationally representative survey conducted in early October 2020 found that 91 percent of parents intended to send their children back to in-person school (Cuccaro et al., 2021). A low dropout rate (3 percent) was also found in another third-party survey of a representative sample of students in Rising Academy schools across Sierra Leone, Liberia, and Ghana, between January–March 2021 (Caballero Montoya et al., 2021).

Only around half of children in Sierra Leone were engaged in any educational activity while schools were closed, according to a nationally representative survey conducted in July 2020. Less than half listened to educational radio, spending on average four hours per week listening to radio lessons (from a maximum possible of 7 hours for grades 1–3 or 5 hours for grade 4–6). 99 percent of parents expected their children to return to school, but only around half expected their children to be promoted to the next grade (Foster, 2020). This re-enrolment was later confirmed in a second survey in November/December 2020, which found that 97–99 percent of previously enrolled students had returned. Actual grade promotion rates were higher than expected by parents, at around 75 percent (Foster, 2021).

Learning outcomes were dire even before the crisis. A 2014 national Early Grade Reading Assessment (EGRA) found that 97 percent of children in class 2 could not read (DSTI Media, 2019). Only 83 percent of children complete primary school (World Bank, 2021).

The programme we study was implemented by the non-government education provider Rising Academies in partnership with the Government of Sierra Leone. Rising Academies launched in Sierra Leone in 2014 and provided emergency education to children who were out of school due to school closures during the Ebola epidemic. Rising Academies manages 157 private and government schools in Sierra Leone, Liberia, and Ghana, and works closely with government in Sierra Leone and Liberia. Public schools managed by Rising Academies in Liberia have been shown to be effective (Romero and Sandefur, 2022, Romero et al., 2020). Prior to the pandemic, Rising Academies had been supporting 25 government primary schools since January 2020 as part of the government’s Education Innovation Challenge programme. Education Innovation Challenge schools are government schools staffed by government teachers, in which one of five non-state operators have been invited to test pedagogical and other innovations with the potential to improve the quality of teaching and learning at scale. The intervention we study took place in these Education Innovation Challenge schools.

The Sierra Leone Ministry of Education broadcast educational content by radio for all grade levels, from lower primary to secondary. The Ministry has had a radio education unit since the end of the civil war in 2002 as a complement to schools (Alghali et al., 2005, Mangesi, 2007), and it broadcast educational radio during Ebola-related school closures. New radio learning content was developed by government and partners (including Rising Academies) specifically for the COVID-19 school closures to replace in-person instruction. Rising Academies produced Mathematics and English lessons for lower and upper primary. The Radio Teaching Programme broadcast Rising’s lower primary lessons three days per week on national radio. Rising Academies also broadcast two hours of Mathematics and English lessons for lower and upper primary every day on six local radio stations (Lamba and Reimers, 2020).

Before the pandemic, 73 percent of all households owned a mobile phone, 55 percent owned a radio, 20 percent owned a television, and 5 percent owned a computer (Statistics Sierra Leone (Stats SL) and ICF, 2020). Government therefore did not offer any distance-learning provision by television or online.

Despite many students having access to a radio, children may miss out on radio instruction simply through limited attention to the time schedule while at home. To encourage participation, Rising Academies sent SMS reminders to students, including (in principle) all households in all treatment arms of our experiment described below. The phone number listed for each student or guardian received a total of 48 SMS messages over 18 weeks, or an average of 2.7 messages per week. Messages were either simple reminders about the time of the education radio broadcast, an exercise to be completed, or a piece of advice for parents. The SMS number was free to respond to, and students were encouraged to reply with their answers. Messages were addressed from Rising Academies.

## 3 Intervention

In addition to their other work contributing to the government’s national radio programme and sending SMS reminders about these programmes, Rising Academies designed and implemented a tutorial phone call intervention, designed to be complementary to the radio programming, for students from the 25 government schools that they were supporting as part of the Education Innovation Challenge. Rising Academies collected around 5,600 phone numbers of students from these schools in the two days prior to school closures in March 2020. Students were then called so that teachers could recap lessons delivered by radio and answer questions. Interaction is critical to learning, such that there are limits to the overall effectiveness of entirely one-way instruction delivered through mass media such as radio. Delivering actual instruction by phone allows for two-way communication, so teachers can check for the understanding of children and adjust instruction in real-time as necessary.

The interventions began on 25th May 2020 and continued through the end of August 2020. Interventions were initially planned to last for 12 weeks from May to July, and were extended into August for a total of 16 weeks of programming. Educational radio programmes were broadcast on national radio and on six local radio stations.

Several studies have shown that the same intervention can have bigger effects when delivered by an NGO than when delivered by government (Bold et al., 2018, Vivalt et al., 2021, Kerwin and Thornton, 2021). We therefore test the same intervention delivered by private school teachers employed by the implementer (Rising Academies) and by public school teachers employed by the government. Students from the public schools in our sample were randomised to be either called by private school teachers employed by Rising Academies, or by government school teachers. As the implementer has more direct influence over its own employees, we expect this version to test the potential of the intervention at high fidelity, and the version with government teachers to give greater insight into the potential for scalability.

The intervention was delivered by 80 private school teachers and 80 government school teachers. Each teacher was assigned an average of 35 students, and that teacher stayed with the same group of students throughout. Each teacher taught one subject (reading or mathematics) and grade level (upper or lower primary) in the phone tutorials. Teachers did not teach their own usual class.

The private school teachers involved in delivering the intervention had been working for Rising Academies for an average of three years. Government school teachers had been introduced to Rising Academies through the “Education Innovation Challenge” government partnership programme that started in January 2020. Both the private and public school teachers continued to receive their normal salary whilst schools were closed. They received phone calling credit to cover the cost of calls. In May 2020 government teachers received a pre-agreed 30 percent pay rise, the largest rise in a decade (Murray, 2020). All teachers at the 25 government schools were invited to participate in the programme. Participating teachers received no additional compensation, over and above their normal salary which was still paid during the pandemic, with the exception of a small bonus. The bonus was to cover work over August when schools would usually be on holiday, and it amounted to around 80,000 Leones per teacher, roughly USD 8. Government data suggested that there were 232 teachers in these schools in 2019, which would imply that around a third chose to participate. As schools were closed, teachers did not have other responsibilities besides making these calls. With the raise in government teachers’ pay, the government and private school teachers in our sample earn similar amounts. The private school teachers have an average of 7 years of experience, half as much as the government school teachers who have an average of 14 years of experience. The private school teachers are more likely to have university education than the government school teachers (Table A.1 in Appendix A).

The intervention aimed to deliver a weekly call to each student from each of their two assigned teachers (one focused on mathematics and one on reading). All households from whom phone numbers were gathered were included in the randomisation. Each call was expected to last for around fifteen minutes. Teachers identified themselves as teachers, and carried out telephone-based tutorials. These tutorials were consistent with the curriculum of the radio programming. The calls reviewed and recapped the material covered in the radio broadcast, following a detailed guide. (Appendix B includes sample evaluation items, and Appendix C includes sample scripts.) Programme monitoring data suggests that private school teachers placed more calls than government school teachers. The average respondent in the private teacher treatment arm received ten out of a maximum possible 16 calls focused on mathematics and nine out of 16 on language. Respondents in the government teacher arm received seven out of 16 planned calls on mathematics and six out of 16 on language. Students may not have received all 16 of the planned calls in part due to difficulties with phone signal, timing of calls, or getting access to a shared family phone. However, the difference in the number of calls received from private school and government school teachers is most likely due to differing incentives facing those employed directly by the implementer.

## 4 Experimental design and econometric specification

**Fig. 1:**
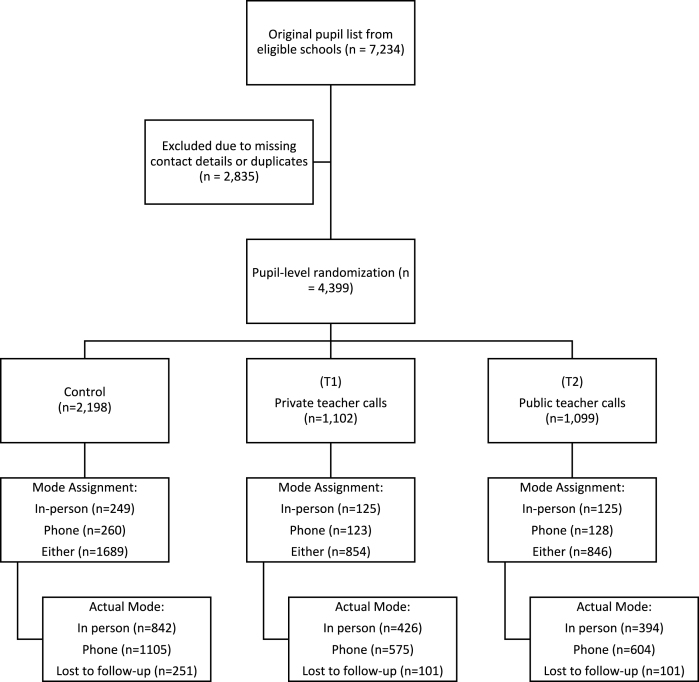
Consort diagram.

We randomised households into a control group or one of the two treatment groups.^2^ Randomisation was stratified according to baseline test scores and grade of students (where baseline data were available).^3^ Within each household with more than one child who had been attending one of the 25 schools, we randomly selected one child to be interviewed in the follow-up survey (although each child with the households was intended to receive the household’s assigned treatment). Students were also cross-randomised to either an in-person interview (499), phone interview (511), or to whichever of these two methods was most convenient (3,389). The number of students at each stage is shown in Fig. 1.

We estimate the following specification: we regress each outcome $Y_{is}$ on an indicator variable for whether the student was assigned to receive calls (from either public or private teachers), and an indicator for whether the student was assigned to receive calls from a public school teacher in particular.

$$Y_{is}=\beta_{0}+\beta_{1}Calls_{i}+\beta_{2}Public_{i}+\gamma X_{i}+Z_{s}+\epsilon_{is}$$

Our coefficients of interest are $\beta_{1}$ and $\beta_{2}$. We also include student controls $X_{i}$ and school fixed effects $Z_{s}$, and calculate robust standard errors.

## 5 Data

### 5.1 Baseline

Prior to school closures, the implementing agency (Rising Academies) collected contact information for 5,566 students from 4,407 households, along with their grade, school, date of birth, and father’s name. For a sub-set of 3034 of these students (in grade 3–6), the implementing agency conducted basic literacy and numeracy assessments adapted from the ASER Centre tools, between 25th February and 20th March 2020.

### 5.2 Interim survey

We conducted a short interim independent survey between 10th and 19th September 2020, shortly after the end of treatment at the end of August. This survey targeted a randomly selected sub-sample of 815 children. Of these, 413 children (51 percent) were able to be tracked (with no statistically significant difference in response rates between treatment groups). The focus of this interim survey was on time spent engaging with distance learning and related educational activities. For parent and for child educational activities, we measure five binary indicators and then calculate an index by taking the first principal component of the five binary items, and standardising this with the control group mean and standard deviation. For parents, this index comprises binary indicators for whether – since schools closed – they had talked about school with their child, read to their child, paid for tutoring, called their teacher, and whether they knew the correct FM frequency for educational radio. For children, this index comprises binary indicators for whether – since schools closed – they had watched educational TV programmes, listened to educational radio programmes, read books, were taught by a parent, and whether they overall spent as much time as their parents would have liked on educational activity.

### 5.3 Endline survey

Schools reopened on 5 October 2020. A key outcome we are interested in is the effect of treatment on re-enrolment. Full enrolment in Sierra Leone typically takes several weeks from the day that school starts, so we began our main survey five weeks after the reopening of school, on Monday (9th November 2020), and ten weeks after the end of the intervention. We collect data on test scores, re-enrolment in school, and time spent on distance learning.

Students were asked to estimate roughly how many minutes they spent per day on all educational activities in a typical week between April and July 2020 while schools were closed. Parents were asked the same question in phone-based surveys.

**Table 1:** All schools’ vs. sample schools’ characteristics .

	All schools	Our sample
	percent	percent
National primary exam pass rate	64	73
Feeding programme	41	80
Recreational facilities	56	20
Electricity	12	8
Drinking water	67	68
Handwashing facilities	66	72
Toilets	74	84
In freetown	11	36

	Mean	Mean

Total enrolment	195.3	290.3
Years in operation	27.5	39.6

N	6,895	25

Note: This table shows descriptive statistics for the schools from our sample and how they compare to all schools nationwide. Data is drawn from the National Examination results and the Ministry of Basic and Senior Secondary Education (MBSSE) Education Management Information System (EMIS) 2019 Annual School Census. A map of school locations is shown in the Fig. A.1.

**Table 2:** Implementation and effects on time use .

	Effect of calls (T1/T2)	Marg effect of Pub. Teach (T2)	Control mean	Obs.
*Outcomes:*				
Parent recalls receiving SMS	0.228${}^{***}$	−0.0546	0.286	406
	(0.0527)	(0.0545)		
Parent recalls receiving calls	0.624${}^{***}$	0.0508	0.0914	406
	(0.0489)	(0.0514)		

*Parent activity:*				
Index (Control mean = 0, sd = 1)	0.268 ${}^{**}$	−0.128	0	406
	(0.112)	(0.113)		
Talks about school	0.0771${}^{*}$	−0.0132	0.824	406
	(0.0398)	(0.0402)		
Reads to child	0.0722	−0.0936	0.378	406
	(0.0630)	(0.0703)		
Pays for tutoring	0.00971	−0.0202	0.523	406
	(0.0623)	(0.0693)		
Calls teacher	0.0755${}^{*}$	−0.00687	0.0984	406
	(0.0425)	(0.0532)		
Knows FM frequency	0.0378${}^{*}$	0.0128	0.332	1,496
	(0.0222)	(0.0238)		

*Child activity:*				
Index (Control mean = 0, sd = 1)	0.288${}^{**}$	−0.171	0	406
	(0.127)	(0.137)		
Educational TV	0.0556	−0.107${}^{**}$	0.202	406
	(0.0511)	(0.0536)		
Educational Radio	0.0869	0.0695	0.570	406
	(0.0576)	(0.0658)		
Reading	0.0669	−0.0206	0.705	406
	(0.0569)	(0.0612)		
Parent teaching	−0.00138	−0.0849	0.389	406
	(0.0627)	(0.0678)		
As much as parent would like	0.0902	−0.0831	0.679	406
	(0.0549)	(0.0638)		

*Time spent on learning:*				
Mins/day (Sep Report)	3.623	−1.033	83.01	406
	(4.897)	(5.501)		
Radio mins/day (Sep Rpt)	5.516	8.205	37.62	406
	(4.722)	(5.767)		
Mins/day (Dec Report)	−2.685	−0.178	81.33	2,288
	(1.793)	(2.010)		
Mins/day (Dec Rpt, Child)	−2.877${}^{*}$	−0.102	83.83	3,952
	(1.463)	(1.674)		

Note: Outcome variables are binary unless otherwise indicated. The parent and child activity indexes are each the first principal component of the subsequent five items listed. Variables with 406 observations are from the small September 2020 interim survey, others are from the full December 2020/January 2021 endline survey. All regressions include school fixed effects and robust standard errors.

### 5.4 Learning assessment

We designed an assessment that could be administered verbally either by phone or in person. We randomised a sub-sample of 499 children to be interviewed in-person.^4^ The objective of that randomisation was to permit a comparison of in-person versus phone assessments; unfortunately, the results of that comparison proved inconclusive; however, we do find that it is feasible to implement phone-based assessments, that phone-based assessments have good internal reliability, and that the mode of assessment does not affect our estimates of treatment effects (Appendix E). In-person surveys took place at schools. We select a combination of items from Early Grade Reading and Mathematics Assessments (EGRA and EGMA), ASER assessments, and items used orally in in-person assessments in urban India (Banerjee et al., 2017b). Parents were reassured that the questions would all remain anonymous, and children should be encouraged to feel comfortable and relaxed. Here we discuss sources of validity evidence for our learning assessment across five areas: content, cognition, coherence, correlation, and consequence (Ho, 2020, American Educational Research Association et al., 2018).

1. **Content:** All of the question items from our assessment are relevant for the content of the tuition that students received. Specifically, we selected items that are similar to questions to be asked by teachers in the scripts for the tutorials. In mathematics this includes counting and simple arithmetic, and in English this includes a test of vocabulary, spelling, and aural comprehension.
2. **Cognition:** We piloted our assessment with a small sample of 32 households to confirm that children responded to the questions in the way that we anticipated. Based on the pilot we updated the assessment to include a definition of words that students were asked to spell.
3. **Coherence:** Items in the mathematics and language assessments have a high level of internal reliability in both in-person and phone samples, and higher inter-item correlation in the phone samples (Table E.1). This suggests that the questions are measuring the same underlying construct (mathematics and language ability). We construct test score outcomes using item-response theory (IRT) (Das and Zajonc, 2010). This allows us to estimate the underlying unobserved traits of mathematical and language ability, while allowing the difficulty and discrimination of individual question items to vary. This is a more conceptually accurate approach than the more common approach of simply giving the percentage of correct answers, which gives the same weight to questions of different difficulty. The method of aggregating test questions can have large implications for estimated effect sizes (Singh, 2015). IRT also allows us to test whether questions have different difficulty and discrimination across the two survey modes (i.e., Differential Item Functioning or DIF). We first estimate a two parameter logistic model with the 12 mathematics items, and a hybrid partial credit and two parameter model for the 11 language items. We then estimate differential item functioning across the two survey modes with logistic regression (Table E.2, Table E.3), following Swaminathan and Rogers (1990).^5^ In order to compare test scores between individuals who were surveyed in person or by phone, we then re-estimate the IRT models, only using the subset of items which appear to perform similarly across mode to link scores across the two assessments. There is differential item functioning (either uniform or non-uniform) for 16 of the 24 individual survey questions between the actual in-person and phone-based survey modes, at the 5 percent level (Table E.2). Comparing responses by randomised mode assignment, there is differential item functioning for only 8 of the 24 questions (Table E.3).
4. **Correlation:** Our assessments are highly correlated with the baseline in-person ASER assessments administered by the programme implementer. This correlation is not statistically significantly different for those assigned to in-person or phone assessment (Table E.4).
5. **Consequence:** Similar assessments to ours have been used in a range of contexts for monitoring school performance. Conducting these assessments by phone holds the potential to substantially reduce the costs of this monitoring, if phone assessments can be shown to be reliable.

### 5.5 Sample characteristics and representativeness

Our study takes place with students from 25 government primary schools in four districts; Western Area Urban (Freetown), Bo, Kailahun, and Kenema. These schools were selected by government to receive pedagogical support from Rising Academies beginning in the 2019/2020 school year as part of the government’s “Education Innovation Challenge”. Several private providers were competitively selected to support different schools. Rising Academies started supporting these schools in January 2020.

Compared to other schools in the country, those in our sample schools are larger, more likely to be in Freetown, and have slightly higher national exam pass rates, but are by no means elite schools. They have similar levels of basic amenities as other schools nationwide, such as electricity (8 percent), drinking water (68 percent), handwashing facilities (72 percent), and toilets (84 percent) (Table 1). Most students were aged between 7 and 17 (with 1 percent of outliers aged between 3 and 20). Control group students in our sample perform comparably on addition and subtraction problems to a recent evaluation of students in grades 1 through 3 in primary schools across 16 districts of Sierra Leone (Montrose International, 2021). Students in our sample perform better on division questions than students in, for example, Botswana, likely because we use simpler division questions (e.g., 9 ÷ 3 rather than 93 ÷ 6) (Angrist et al., 2020b).

**Table 3:** Effect of treatment on test scores .

	Maths		Language	
	(1)	(2)	(3)	(4)
Effect of calls (T1 or T2)	0.006	−0.008	−0.025	−0.027
	(0.038)	(0.034)	(0.038)	(0.034)
Marginal effect of gov. teachers (T2)	−0.009	0.001	0.033	0.032
	(0.044)	(0.039)	(0.043)	(0.039)
School fixed effects		Yes		Yes
Controls		Yes		Yes

Lee Bounds				
(Lower)	−0.057	−0.057	−0.125	−0.125
(Upper)	0.118	0.118	0.149	0.149
Observations	3,946	3,946	3,946	3,946

Notes: Robust standard errors in parentheses.

Controls include student age, sex, grade, and baseline test scores.

* p < 0.10, ** p < 0.05, *** p < 0.01.

### 5.6 Balance and attrition

Randomisation was stratified by student sex, grade, and baseline test scores. A balance test shows that there is no statistically significant difference in mean values for these variables across treatment groups (Table A.2).

Overall we were able to track 90 percent of students. Just over half of these were surveyed by phone. Data collection was conducted sequentially, first calling all numbers (except the 500 student sub-sample randomly reserved for in-person surveying), before moving to in-person tracking. This allows us to show how the characteristics of those able to be tracked by phone differs to those we could track in person, as well as the characteristics of those we were not able to track at all. None of the treatment arms are statistically significantly correlated with tracking by phone, but students were less likely to be reached by phone if they lived outside of Freetown, and if their parents had not completed any school. This suggests that surveys conducted entirely by phone are likely to under-represent the most marginalised. Overall, students who received tutoring calls were marginally more likely to be tracked. Students in Grade Six were 8 percentage points less likely to be found overall, and in Freetown 7 percentage points less likely (Table A.3).

Our response rate compares favourably to purely phone-based assessments (Angrist et al., 2020b, Schueler and Rodriguez-Segura, 2021, Etang and Himelein, 2020), highlighting the importance of in-person tracking to minimise attrition.

### 5.7 Qualitative interviews

To better understand how the programme was perceived by participants, we commissioned a parallel qualitative study with pupils, parents, and teachers. This included a total of 23 focus group discussions with both treatment and control group pupils and parents, at 11 of the 25 programme schools, spread across all four districts (Sam-Kpakra, 2021). It also included 5 interviews with public and private school teachers.

## 6 Results

### 6.1 Implementation

Administrative data on SMS messaging shows that over 92 percent of phone numbers received all of the planned SMS messages (three per week). Parents in the tutoring call groups were 63 percentage points more likely to report receiving a call from a teacher, and 25 percentage points more likely to report receiving SMS messages. Out of a maximum of 16 potential calls in each subject, students in the private school teacher group received an average of 10 calls in mathematics and 9 calls in language, compared to students in the government school teacher group who received an average of 7 calls in mathematics and 6 in language. Parents reported that calls lasted an average of 22 minutes, and that children spent on average just over one hour per day listening to educational radio.

We observe a 0.27 standard deviation effect of receiving calls on the index of parent educational activity (Table 2). This comprises a 8 percentage point effect on the probability of a parent talking to their child about school, 7 point increase in reading to their child (though this estimate is statistically insignificant), 8 point increase in the parent having called a teacher themself, and 4 point increase in knowing the correct educational radio frequency. The coefficient on paying for tutoring was a statistically insignificant 1 percentage point.

We also see a 0.29 standard deviation effect on the index of child educational activity (Table 2). None of the individual activities comprising this index are themselves statistically significantly moved by treatment, though the coefficients are positive. These activities are watching educational TV (6 percentage points), educational radio (9 percentage points), reading (7 percentage points), being taught by a parent (−0.1 percentage points) and spending as much time as their parent would like (9 percentage points).

We see no statistically significant differences in time spent on learning, retrospectively reported by either parent or child. Of parents who reported that their children spent less time on education than they would have liked (31 percent of parents), the most common reason given for this was “no motivation or interest”. The coefficient on calls is equivalent to a 10 percentage point increase in the probability of listening to educational radio at all, though this estimate is only marginally statistically significant at the 10 percent level.

**Fig. A.1:**
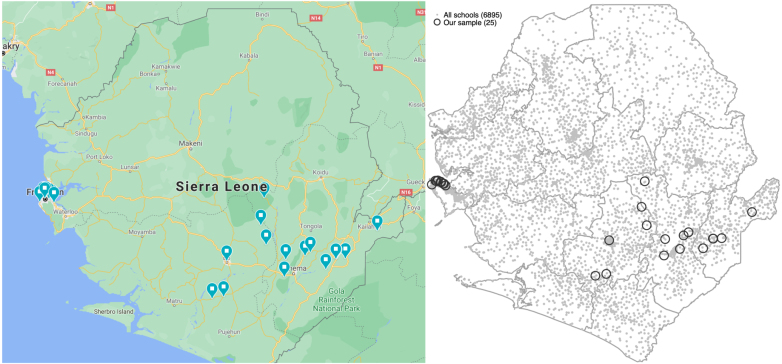
Maps of experimental schools. Note: This figure shows the location of the 25 schools included in our sample. They are located four districts: Western Area Urban (Freetown), Bo, Kailahun, and Kenema.

**Table A.1:** Intervention teacher characteristics.

	Private	Government
Salary (median, Leones)	1,100,000	1,000,000
Experience (mean years, total)	6.5	14.2
Number of Teachers, by Education Level		
- Secondary School	5	5
- Teaching Certificate	4	33
- Higher Teaching Certificate	21	28
- BSc (Education)	4	1
- BSc (Other)	21	5
- Other	24	7
- Total	79	79

Note: This table shows descriptive statistics for private and government school teachers who delivered the tutoring call intervention, based on a survey conducted by the implementing organisation.

**Table A.2:** Baseline balance .

Variable	(1)	(2)	(3)	F-test
	Control	T1 Pr Tchr	T2 Go Tchr	for joint
	Mean/SE	Mean/SE	Mean/SE	orthogonality
Age	11.43 (0.03)	11.37 (0.05)	11.42 (0.05)	0.67
Male	0.48 (0.01)	0.48 (0.02)	0.50 (0.02)	0.62
Baseline grade	3.54 (0.04)	3.54 (0.05)	3.53 (0.05)	0.97
Baseline test score	0.02 (0.02)	0.01 (0.02)	0.01 (0.02)	1.00
N	2,198	1,102	1,099	

*Notes*: P-values reported for F-test, which is estimated with school fixed effects. Standard errors reported in parentheses. Total observations is 4,399.

**Table A.3:** Predictors of attrition .

	Found by phone		Found at all	
	(1)	(2)	(3)	(4)
Effect of calls (T1 or T2)	0.008	0.012	0.023${}^{**}$	0.022${}^{**}$
	(0.019)	(0.019)	(0.011)	(0.011)
Marginal effect of gov. teachers (T2)	0.031	0.024	−0.000	0.001
	(0.022)	(0.022)	(0.012)	(0.012)
Age		−0.002		0.004
		(0.005)		(0.003)
Assigned to in-person test		−0.227${}^{***}$		−0.034${}^{**}$
		(0.026)		(0.017)
In Grade 6		0.120${}^{***}$		−0.076${}^{***}$
		(0.023)		(0.016)
Freetown		0.139${}^{***}$		−0.065${}^{***}$
		(0.017)		(0.009)
Baseline test score		0.017		−0.003
		(0.011)		(0.007)
Parent: Primary		0.073${}^{***}$		
		(0.021)		
Parent: Secondary		0.064${}^{***}$		
		(0.020)		
Parent: Tertiary		0.080${}^{***}$		
		(0.025)		

Outcome mean	0.58	0.59	0.90	0.90
Observations	3,953	3,888	4,399	4,399

Notes: Robust standard errors in parentheses.

* p < 0.10, ** p < 0.05, *** p < 0.01.

**Table A.4:** Effects on individual Maths items .

	Counting		Addition			Subtraction			Division		Multiplication	
	(1)	(2)	(3)	(4)	(5)	(6)	(7)	(8)	(9)	(10)	(11)	(12)
Effect of calls (T1 or T2)	0.018	0.013	0.045${}^{***}$	0.018	0.006	0.015	0.028${}^{*}$	−0.015	0.015	0.001	−0.006	−0.001
	(0.011)	(0.012)	(0.016)	(0.017)	(0.016)	(0.018)	(0.017)	(0.018)	(0.016)	(0.017)	(0.017)	(0.018)
Mrgnl effect gov tchrs (T2)	0.003	0.007	−0.022	−0.023	−0.005	0.015	−0.012	0.020	−0.003	0.014	0.017	0.005
	(0.013)	(0.014)	(0.018)	(0.019)	(0.019)	(0.020)	(0.019)	(0.021)	(0.018)	(0.020)	(0.020)	(0.020)
Assigned to in-person test	0.004	0.006	−0.010	0.008	−0.020	−0.045${}^{*}$	0.004	−0.024	−0.037	−0.014	0.001	0.012
	(0.016)	(0.017)	(0.023)	(0.024)	(0.024)	(0.025)	(0.024)	(0.026)	(0.022)	(0.025)	(0.025)	(0.025)
School FE	Yes	Yes	Yes	Yes	Yes	Yes	Yes	Yes	Yes	Yes	Yes	Yes
Controls	Yes	Yes	Yes	Yes	Yes	Yes	Yes	Yes	Yes	Yes	Yes	Yes

Control mean	0.88	0.87	0.71	0.67	0.31	0.55	0.32	0.54	0.74	0.64	0.43	0.49
Observations	4,399	4,399	4,399	4,399	4,399	4,399	4,399	4,399	4,399	4,399	4,399	4,399
R${}^{2}$	0.049	0.040	0.085	0.091	0.099	0.091	0.085	0.078	0.062	0.073	0.099	0.110

Notes: The dependent variable in each column is a binary indicator for whether the student got that individual item correct (1) or incorrect (0).

The test includes 2 counting items, 3 addition, 3 subtraction, 2 division, and 2 multiplication. All individual items are shown in Appendix B.

Robust standard errors in parentheses. Controls include student age, sex, grade, and baseline test scores. ${}^{*}$p<0.10, ${}^{**}$p<0.05, ${}^{***}$p<0.01.

**Table A.5:** Effects on individual Language items .

	Vocabulary		Spelling			Comprehension						
	(1)	(2)	(3)	(4)	(5)	(6)	(7)	(8)	(9)	(10)	(11)	(12)
Effect of calls (T1 or T2)	−0.088	0.017	0.014	0.007	0.030${}^{**}$	0.006	0.022	−0.001	0.022	−0.003	−0.016	0.008
	(0.086)	(0.096)	(0.012)	(0.017)	(0.015)	(0.014)	(0.017)	(0.017)	(0.015)	(0.017)	(0.017)	(0.018)
Mrgnl effect gov tchrs (T2)	−0.050	−0.148	0.005	0.015	0.001	0.011	0.015	0.025	0.014	0.030	0.047${}^{**}$	0.008
	(0.099)	(0.110)	(0.014)	(0.019)	(0.018)	(0.017)	(0.020)	(0.020)	(0.017)	(0.019)	(0.020)	(0.020)
Assigned to in-person test	−0.157	−0.207	0.009	−0.051${}^{**}$	−0.013	−0.015	0.004	0.001	−0.002	−0.009	0.039	0.005
	(0.128)	(0.142)	(0.017)	(0.024)	(0.022)	(0.021)	(0.025)	(0.025)	(0.022)	(0.024)	(0.025)	(0.025)
School FE	Yes	Yes	Yes	Yes	Yes	Yes	Yes	Yes	Yes	Yes	Yes	Yes
Controls	Yes	Yes	Yes	Yes	Yes	Yes	Yes	Yes	Yes	Yes	Yes	Yes

Control mean	7.20	5.67	0.87	0.65	0.75	0.79	0.58	0.63	0.75	0.66	0.41	0.55
Observations	3,953	3,953	4,399	4,399	4,399	4,399	4,399	4,399	4,399	4,399	4,399	4,399
R${}^{2}$	0.169	0.167	0.050	0.105	0.076	0.074	0.088	0.078	0.089	0.082	0.089	0.100

Notes: The dependent variable in each column is a binary indicator for whether the student got that individual item correct (1) or incorrect (0).

The test includes 2 vocabulary items, 3 spelling, and 7 comprehension. All individual items are shown in Appendix B.

Robust standard errors in parentheses. Controls include student age, sex, grade, and baseline test scores. ${}^{*}$p<0.10, ${}^{**}$p<0.05, ${}^{***}$p<0.01.

**Table A.6:** Effects of treatments on IRT vs. Simple total test scores .

	Maths		Language	
	(1)	(2)	(3)	(4)
	IRT	Total	IRT	Total
Effect of calls (T1 or T2)	−0.008	−0.011	−0.027	−0.025
	(0.034)	(0.034)	(0.034)	(0.034)
Mrgnl effect gov tchrs (T2)	0.000	−0.003	0.032	0.045
	(0.039)	(0.039)	(0.039)	(0.039)
School FE	Yes	Yes	Yes	Yes
Controls	Yes	Yes	Yes	Yes

Observations	3,946	3,946	3,946	3,946
R${}^{2}$	0.213	0.216	0.210	0.235

Notes: Robust standard errors in parentheses.

Controls include student age, sex, grade, and baseline test scores.

${}^{*}$p<0.10, ${}^{**}$p<0.05, ${}^{***}$p<0.01.

**Table A.7:** Effect of treatment on test scores, by sex .

	Maths		Language	
	(1)	(2)	(3)	(4)
	Boys	Girls	Boys	Girls
Effect of calls (T1 or T2)	−0.025	0.008	−0.022	−0.044
	(0.048)	(0.049)	(0.051)	(0.048)
Marginal effect of gov. teachers (T2)	−0.034	0.036	0.022	0.045
	(0.055)	(0.055)	(0.057)	(0.053)
School fixed effects	Yes	Yes	Yes	Yes
Controls	Yes	Yes	Yes	Yes

Observations	1,916	2,029	1,916	2,029

Notes: Robust standard errors in parentheses.

Controls include student age, grade, and baseline test scores.

* p < 0.10, ** p < 0.05, *** p < 0.01.

### 6.2 Outcomes

Almost all (99.7 percent) of respondents in our sample report that their child has re-enrolled in school and attended in the last week, so we do not see any difference in this outcome by treatment status.

There is no effect of tutoring calls on mathematics or language test scores, by either private or government teachers (Table 3). With the upper bound of the 95 percent confidence interval around the estimate of the marginal effect of calls, we can rule out effects larger than 0.08 standard deviations in mathematics and 0.05 standard deviations in language–or 0.12 standard deviations and 0.15 standard deviations using Lee Bounds to bound possible remaining bias due to attrition. Coefficients on other covariates have the expected sign effect and significance.

### 6.3 Robustness and heterogeneity

Looking at individual test question items, we see a small statistically significant effect for just two of the 12 mathematics items (0.03–0.05 standard deviations) and for one of the 11 language items (0.03 standard deviations, see Table A.4, Table A.5). Results are little changed when aggregating test items using item-response theory estimates or a simple total of correct questions (Table A.6), and when testing in-person or by phone (Table E.5).

Across sub-groups, we see similarly insignificant results for literacy and mathematics for girls and for boys (Table A.7). We also see no statistically significant interactions between treatment and student sex, grade, parent education, or baseline test scores (Table A.8). We observe no difference in effects by the intensity of treatment (number of calls actually successfully placed) (Table A.9).

### 6.4 Costs

We analyse programme cost data following a format outlined by the World Bank, designed to allow comparability of costs across countries (Holla, 2019). The average cost of the SMS treatment is $2 per participant, and the average cost of the tutoring call treatment is $40 per participant. This average cost includes phone charges, teacher salaries, and management staff time in the design and oversight of the programme, with all cost components disaggregated as far as possible.

### 6.5 Explaining null effects

In this section we discuss two possible explanations for the observed null effects; implementation challenges, and spillovers.

Qualitative interviews with children found a number of reasons that could explain poor overall performance. Some found the timing of calls challenging. For example, if parents had to work during the day then a child may have either had to try and take the tuition call at a noisy and distracting location such as a marketplace, or take the call in the evening, when they were tired. Some more rural locations had challenges with mobile phone network and electricity supply. These explanations fit with the overall pattern of low engagement with the programme, with only half of the scheduled calls being actually placed, and no significant effect on overall time spent in educational activity.

In some areas, some pupils reported that teachers only spoke Krio and English, and not the local language (Mende). Some pupils mentioned that they struggled without being able to see their teacher writing on the blackboard as they were used to.

*“Sometimes when the teacher called my father will not be at home at the moment and he will ask the teacher to call at night and when the teacher calls at night, I won’t be able to have total understanding because at that time I had started becoming sleepy, I will just pretend that I understood the lesson but in actual sense I do not.*” (Primary School Pupil, Western Urban District)

**Table A.8:** Heterogeneous effects on Maths scores .

	(1)	(2)	(3)	(4)
Effect of calls (T1 or T2)	0.027	−0.019	−0.065	−0.008
	(0.040)	(0.044)	(0.071)	(0.028)
Male × Treat	−0.071			
	(0.056)			
Parent education × Treat		0.014		
		(0.026)		
BL grade × Treat			0.016	
			(0.017)	
BL test scores × Treat				−0.001
				(0.034)
School FE	Yes	Yes	Yes	Yes
Controls	Yes	Yes	Yes	Yes

Observations	3,946	3,881	3,946	3,946
R${}^{2}$	0.214	0.229	0.214	0.213

Notes: Robust standard errors in parentheses.

Controls include student age, sex, grade, and baseline test scores.

${}^{*}$p<0.10, ${}^{**}$p<0.05, ${}^{***}$p<0.01.

**Table A.9:** Effect of treatment intensity (Number of Calls) .

	Maths			Language		
	(1)	(2)	(3)	(4)	(5)	(6)
	OLS	OLS	IV	OLS	OLS	IV
Number of Maths calls	0.012${}^{***}$	0.003	−0.000			
	(0.002)	(0.002)	(0.003)			
Number of language calls				0.002	−0.001	0.002
				(0.002)	(0.002)	(0.004)
School FE	Yes	Yes		Yes	Yes	
Controls		Yes	Yes		Yes	Yes

Observations	3,946	3,946	3,946	3,946	3,946	3,946
R${}^{2}$	0.052	0.214	0.172	0.103	0.210	0.122

Notes: In the IV estimates the number of calls is instrumented for by treatment status.

Robust standard errors in parentheses.

Controls include student age, sex, grade, and baseline test scores.

${}^{*}$p<0.10, ${}^{**}$p<0.05, ${}^{***}$p<0.01.

A second possibility is that the programme was in fact effective, and our estimates are biased by positive spillovers. Qualitative interviews raised some concerns about possible spill-overs across groups, with several pupils and parents from treated households noted that they invited friends and neighbours to listen to the tuition call together.

*“My child was not fortunate to be part of the mobile phone teaching programme. But fortunately, one of his friends invited him as he was part of the mobile phone teaching programme organised by Rising Academy”.* (Parent, Kailahun District)

While these narratives suggest some control pupils may have been exposed to the programme, we provide two pieces of evidence that suggest this exposure did not lead to positive spillovers and hence do not explain our null results. First, there is substantial variation in the number of treated peers that each child had, and therefore the likelihood that they may have been exposed to spillovers. We run a series of regressions in which we test for the presence of spillovers (Appendix F). We include either the share of treated peers and that share interacted with treatment (Table F.1 for mathematics and Table F.2 for language) or the number of treated peers – controlling for the total number of peers – together with an interaction between the number and treatment (Table F.3 for mathematics and Table F.4 for language). In each case, we define the peer group in four different ways: the entire school (Column 1), upper or lower primary (as the radio programming was divided into those two groups — Column 2), peers in the same or adjacent grades (Column 3), or peers only in the same grade (Column 4). Simple peer effects on control group students have negative signs for all specifications except those that treat the entire school as peers, arguably the least likely peers since the content being covered in grades far removed from each other is different (these are also the only specifications that cannot control for school fixed effects).^6^ Thus, it is unlikely that positive peer effects are driving our results. If we calculate the direct treatment effect – adjusted for the inclusion of peer effects – i.e., the linear combination of treatment, treatment interacted with the share or number of treated peers, and treatment interacted with the overall size of the peer group, the treatment effect remains statistically insignificant in all cases, usually with negative coefficients. Alternatively, a “total” treatment effect could also include the effect of spillovers independent of individual treatment (i.e., the coefficient on the share of treated peers). Across 12 specifications, only one of these coefficients is significant, and that – again – is only when treating the entire school as the peer group and omitting school fixed effects.

Second, we might expect a successful programme with a high degree of spillover to be reflected in improved overall school performance. Thus we compare overall national primary exam pass rates before the programme (2019) and after the programme (2020). We have data only for schools in Freetown. This leaves us with 8 schools in our study, and 259 other schools. Overall we see no statistically significant difference in 2020 pass rates between study schools and other government primary schools in Freetown, including when adjusting for prior (2019) pass rates, enrolment, and chiefdom within Freetown (Table F.5).

Taken together, we do not find any consistent evidence that positive spillovers are likely driving our null results.

**Fig. E.1:**
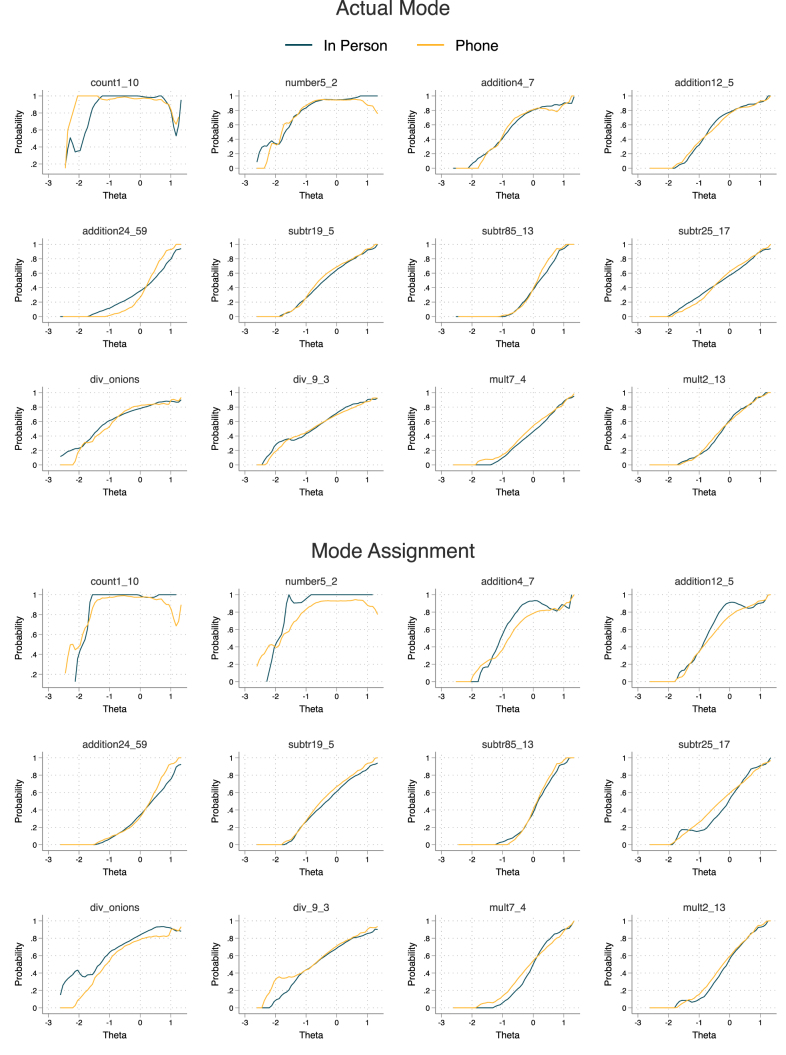
Differential Item Functioning (DIF) - Mathematics. Note: This figures graphs the probability of answering each question correctly, by estimated ability (Theta), by survey mode.

**Fig. E.2:**
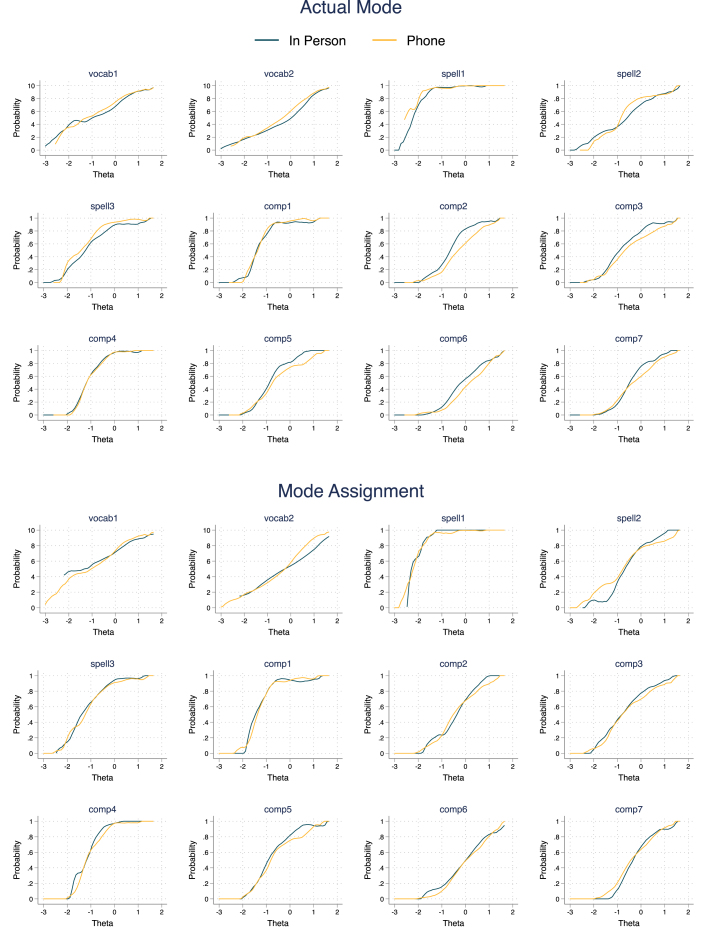
Differential Item Functioning (DIF) - Language. Note: This figures graphs the probability of answering each question correctly, by estimated ability (Theta), by survey mode.

**Fig. E.3:**
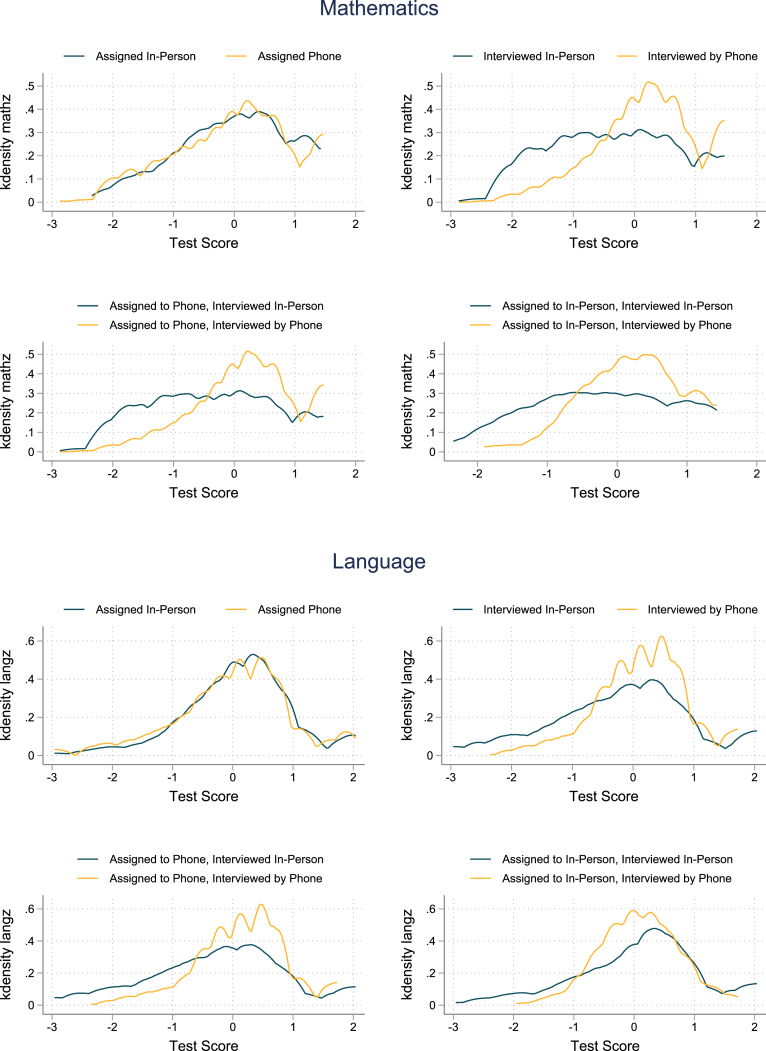
Test score distribution by survey mode. Note: This figure shows the distribution of test scores by survey mode.

## 7 Conclusion

In this paper we tested the effect of live tutoring calls from teachers designed to complement distance learning delivered by radio. We find a small positive effect on engagement with education, but no effect on mathematics and language test scores. We do not see any effect on school re-enrolment, as over 99 percent of respondents re-enrolled (regardless of treatment status).

One limitation to this study is the focus on learning outcomes. Another component of the radio programming and SMS reminders was around improving parenting practices designed to improve child well-being, which we did not measure as an outcome.

While most countries around the world have re-opened their schools, surges of COVID-19 cases may lead to further closures, and future adverse events will lead to school closures in individual countries. This study suggests a need for further experimentation in terms of how to help students stay engaged and learning when schools close. Furthermore, our substantial differences across modes of assessments (phone versus in-person) suggest the need for more research if phone-based assessments are to be a viable tool for measuring student learning.

## Figures and Tables

**Table E.1:** Phone assessment reliability.

	Alpha	Omega	Correlation	N (Items)	N (Students)
In-person (Maths)	0.86	0.87	0.34	12	1,665
Phone (Maths)	0.93	0.92	0.52	12	2,734
In-person (Language)	0.87	0.87	0.35	12	1,665
Phone (Language)	0.90	0.91	0.44	12	2,734

Note: This table shows Cronbach’s Alpha, McDonald’s omega, and the inter-item correlation for the sub-samples of the data tested in-person and by phone.

**Table E.2:** Differential item functioning: Actual mode.

	Non-Uniform		Uniform	
	Chi2	Prob.	Chi2	Prob.
count1_10	18.18	0.00	0.78	0.38
number5_2	0.17	0.68	0.06	0.80
addition4_7	7.02	0.01	3.05	0.08
addition12_5	0.09	0.77	4.56	0.03
addition24_59	50.24	0.00	49.03	0.00
subtr19_5	10.93	0.00	0.15	0.70
subtr85_13	9.81	0.00	6.16	0.01
subtr25_17	1.17	0.28	0.07	0.80
div_onions	2.34	0.13	3.16	0.08
div_9_3	0.02	0.88	7.45	0.01
mult7_4	11.14	0.00	11.34	0.00
mult2_13	0.88	0.35	6.31	0.01
vocab1	29.06	0.00	42.65	0.00
vocab2	10.92	0.00	0.41	0.52
spell1	3.23	0.07	0.95	0.33
spell2	2.94	0.09	26.25	0.00
spell3	3.18	0.07	34.83	0.00
comp1	1.05	0.31	2.59	0.11
comp2	3.97	0.05	147.88	0.00
comp3	8.47	0.00	38.41	0.00
comp4	0.01	0.94	2.07	0.15
comp5	20.02	0.00	35.67	0.00
comp6	7.48	0.01	112.34	0.00
comp7	26.39	0.00	34.27	0.00

**Table E.3:** Differential item functioning: Assigned mode.

	Non-Uniform		Uniform	
	Chi2	Prob.	Chi2	Prob.
count1_10	2.52	0.11	0.99	0.32
number5_2	0.04	0.85	11.49	0.00
addition4_7	0.91	0.34	3.06	0.08
addition12_5	0.13	0.72	9.94	0.00
addition24_59	0.04	0.84	0.86	0.35
subtr19_5	0.11	0.74	5.76	0.02
subtr85_13	0.95	0.33	5.66	0.02
subtr25_17	0.34	0.56	2.10	0.15
div_onions	0.86	0.35	4.28	0.04
div_9_3	0.32	0.57	2.81	0.09
mult7_4	0.65	0.42	0.46	0.50
mult2_13	0.00	0.99	0.01	0.94
vocab1	0.97	0.33	1.39	0.24
vocab2	0.01	0.94	1.30	0.25
spell1	2.44	0.12	1.22	0.27
spell2	2.93	0.09	5.91	0.02
spell3	0.06	0.81	0.14	0.70
comp1	7.14	0.01	0.92	0.34
comp2	0.52	0.47	0.74	0.39
comp3	0.00	0.98	0.36	0.55
comp4	0.36	0.55	1.27	0.26
comp5	0.39	0.53	1.09	0.30
comp6	0.69	0.41	13.23	0.00
comp7	2.50	0.11	0.03	0.87

**Table E.4:** Correlation between baseline and endline tests .

	(1)	(2)	(3)	(4)
	Maths	Language	Maths	Language
Assigned to in-person test	−0.071	0.44		
	(0.062)	(0.133)		
Baseline maths	0.235***		0.164***	
	(0.019)		(0.021)	
Baseline maths × In-person (Assigned)	0.056			
	(0.052)			
Baseline literacy		0.134***		0.067***
		(0.018)		(0.021)
Baseline literacy × In-person (Assigned)		−0.009		
		(0.047)		
In-person (Actual)			−0.538***	−0.500***
			(0.034)	(0.083)
Baseline maths × In-person (Actual)			0.178***	
			(0.036)	
Baseline literacy × In-person (Actual)				0.165***
				(0.033)
School fixed effects	Yes	Yes	Yes	Yes

Observations	3,946	2,389	3,946	2,389
R${}^{2}$	0.090	0.130	0.146	0.144

Notes: Robust standard errors in parentheses.

* p<0.10, ** p<0.05, *** p<0.01.

**Table E.5:** Effect of treatment on test scores .

	Maths			
	(1)	(2)	(3)	(4)
Effect of calls (T1 or T2)	−0.032	0.020	−0.007	0.098
	(0.040)	(0.122)	(0.062)	(0.155)
Marginal effect of gov. teachers (T2)	0.005	−0.018	0.011	−0.139
	(0.046)	(0.142)	(0.073)	(0.165)
School fixed effects	Yes	Yes	Yes	Yes
Controls	Yes	Yes	Yes	Yes

Observations	2,098	186	1,429	233

	Language			
	(1)	(2)	(3)	(4)
Effect of calls (T1 or T2)	0.012	0.025	−0.050	−0.221
	(0.038)	(0.132)	(0.067)	(0.150)
Marginal effect of gov. teachers (T2)	0.045	0.073	−0.001	0.107
	(0.043)	(0.138)	(0.075)	(0.160)
School fixed effects	Yes	Yes	Yes	Yes
Controls	Yes	Yes	Yes	Yes

Observations	2,098	186	1,429	233

Notes: Robust standard errors in parentheses.

Controls include student age, sex, grade, and baseline test scores.

* p<0.10, ** p<0.05, *** p<0.01.

Students in col 1 were randomly assigned to phone and interviewed by phone.

Students in col 2 were randomly assigned to in-person but interviewed by phone.

Students in col 3 were randomly assigned to phone but interviewed in-person.

Students in col 4 were randomly assigned to in-person and interviewed in-person.

**Table E.6:** Effects of survey mode on test scores (IV) .

	Maths (OLS)		IV 1st stage		Maths (IV)	
	(1)	(2)	(3)	(4)	(5)	(6)
Assigned to in-person test			0.151${}^{***}$	0.111${}^{***}$		
			(0.026)	(0.028)		
In-person (Actual)	−0.505${}^{***}$	−0.435${}^{***}$			0.396	−0.284
	(0.032)	(0.030)			(0.347)	(0.409)
School fixed effects		Yes		Yes		Yes
Controls		Yes		Yes		Yes

F-Stat			34.6	10.4		
Observations	3,946	3,946	3,946	3,946	3,946	3,946

	Language (OLS)		IV 1st stage		Language (IV)	
	(1)	(2)	(3)	(4)	(5)	(6)
Assigned to in-person test			0.151${}^{***}$	0.111${}^{***}$		
			(0.026)	(0.028)		
In-person (Actual)	−0.320${}^{***}$	−0.200${}^{***}$			0.774${}^{**}$	−0.150
	(0.033)	(0.031)			(0.343)	(0.417)
School fixed effects		Yes		Yes		Yes
Controls		Yes		Yes		Yes

F-Stat			34.6	10.4		
Observations	3,946	3,946	3,946	3,946	3,946	3,946

Notes: Robust standard errors in parentheses.

The outcome in columns (3) and (4) is whether the individual was actually surveyed in person.

Controls include student age, sex, grade, and baseline test scores.

**Table E.7:** Effects of survey mode (Actual) on Maths scores (ITT) .

	Assigned			Actual		
	(1)	(2)	(3)	(4)	(5)	(6)
	All	Phone	In-person	All	Phone	In-person
Effect of calls (T1 or T2)	−0.019	−0.019	0.055	−0.017	−0.024	0.007
	(0.036)	(0.036)	(0.100)	(0.039)	(0.038)	(0.057)
Mrgnl effect gov tchrs (T2)	0.007	0.008	−0.051	−0.011	−0.002	−0.008
	(0.042)	(0.042)	(0.113)	(0.044)	(0.043)	(0.067)
Assigned to in-person test	−0.072					
	(0.067)					
Calls × In-person	0.097					
	(0.105)					
Calls (Gov) × In-person	−0.062					
	(0.119)					
In-person (Actual)				−0.443${}^{***}$		
				(0.042)		
Calls × In-person				0.015		
				(0.070)		
Calls (Gov) × In-person				−0.001		
				(0.081)		
School FE	Yes	Yes	Yes	Yes	Yes	Yes
Controls	Yes	Yes	Yes	Yes	Yes	Yes

Observations	3,946	3,527	419	3,946	2,284	1,662
R${}^{2}$	0.214	0.212	0.265	0.256	0.233	0.242

Notes: Robust standard errors in parentheses.

Controls include student age, sex, grade, and baseline test scores.

${}^{*}$p<0.10, ${}^{**}$p<0.05, ${}^{***}$p<0.01.

**Table E.8:** Effects of survey mode (Assignment) on Maths scores, by parent education .

	(1)	(2)	(3)	(4)	(5)
	None	Primary	Secondary	Tertiary	All
Effect of calls (T1 or T2)	−0.026	−0.024	0.060	−0.054	−0.002
	(0.049)	(0.061)	(0.050)	(0.070)	(0.028)
In-person (Assigned)	−0.057	0.087	−0.242${}^{***}$	0.107	0.137
	(0.093)	(0.118)	(0.080)	(0.099)	(0.084)
*Parent education:*					
Primary					0.216${}^{***}$
					(0.041)
Secondary					0.307${}^{***}$
					(0.039)
Tertiary					0.459${}^{***}$
					(0.048)
In-person (Assigned) × Primary					−0.025
					(0.133)
In-person (Assigned) × Secondary					−0.478${}^{***}$
					(0.109)
In-person (Assigned) × Tertiary					−0.111
					(0.119)
School FE	Yes	Yes	Yes	Yes	Yes
Controls	Yes	Yes	Yes	Yes	Yes

Observations	1,306	864	1,162	546	3,881
R${}^{2}$	0.233	0.258	0.229	0.267	0.234

Notes: Robust standard errors in parentheses.

Controls include student age, sex, grade, and baseline test scores.

${}^{*}$p<0.10, ${}^{**}$p<0.05, ${}^{***}$p<0.01.

**Table E.9:** Effects of survey mode (Actual) on Maths scores, by parent education .

	(1)	(2)	(3)	(4)	(5)
	None	Primary	Secondary	Tertiary	All
Effect of calls (T1 or T2)	−0.045	−0.014	0.060	−0.056	−0.010
	(0.047)	(0.060)	(0.050)	(0.070)	(0.027)
In-person (Actual)	−0.473${}^{***}$	−0.349${}^{***}$	−0.302${}^{***}$	−0.189${}^{**}$	−0.505${}^{***}$
	(0.052)	(0.067)	(0.058)	(0.083)	(0.050)
*Parent education:*					
Primary					0.136${}^{***}$
					(0.044)
Secondary					0.176${}^{***}$
					(0.042)
Tertiary					0.308${}^{***}$
					(0.049)
In-person (Actual) × Primary					0.130
					(0.080)
In-person (Actual) × Secondary					0.133${}^{*}$
					(0.074)
In-person (Actual) × Tertiary					0.299${}^{***}$
					(0.092)
School FE	Yes	Yes	Yes	Yes	Yes
Controls	Yes	Yes	Yes	Yes	Yes

Observations	1,306	864	1,162	546	3,881
R${}^{2}$	0.280	0.284	0.242	0.274	0.267

Notes: Robust standard errors in parentheses.

Controls include student age, sex, grade, and baseline test scores.

${}^{*}$p<0.10, ${}^{**}$p<0.05, ${}^{***}$p<0.01.

**Figure:**
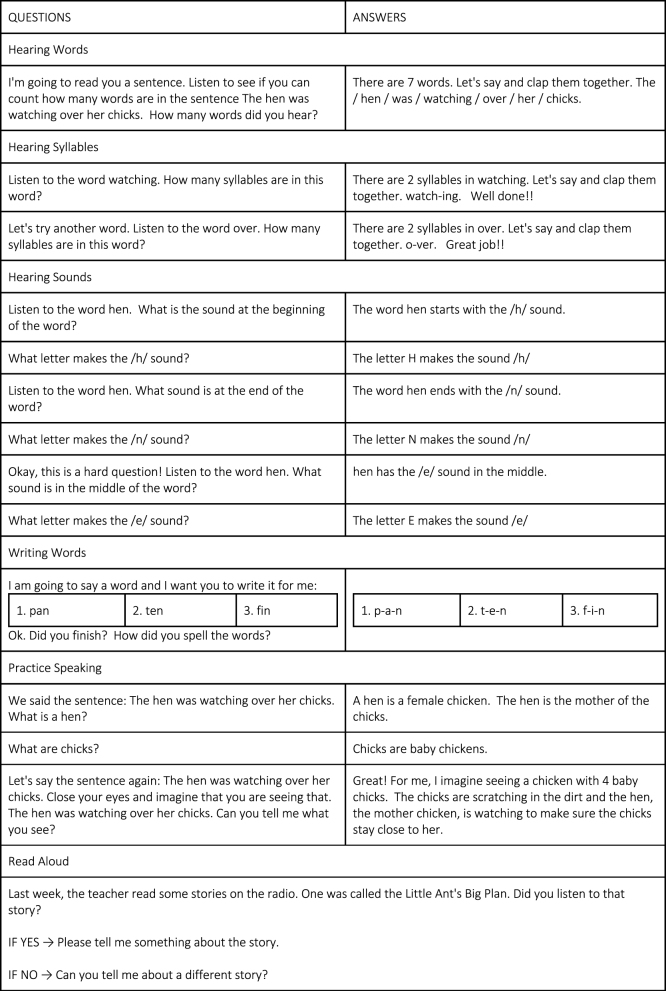


**Figure:**
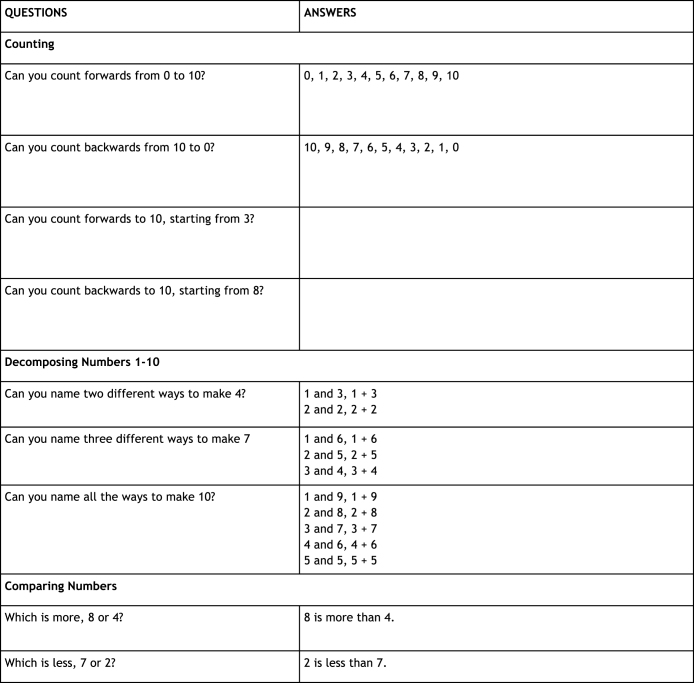


**Table F.1:** Heterogeneous effects by share of treated peers (Maths) .

	(1)	(2)	(3)	(4)
	School	Upper/ Lower	Adj grades	Grade
Treatment	−0.652	−0.101	−0.199	−0.079
	(0.485)	(0.294)	(0.307)	(0.182)
Share of treated peers	0.725	−0.976${}^{***}$	−0.958${}^{**}$	−0.542${}^{**}$
	(0.663)	(0.377)	(0.449)	(0.232)
Treatment × Share	1.224	0.093	0.299	0.059
	(0.925)	(0.558)	(0.582)	(0.330)
Peer group size	−0.000	0.001	0.002${}^{***}$	0.003${}^{**}$
	(0.000)	(0.001)	(0.001)	(0.001)
Treatment × Peer group size	0.000	0.000	0.000	0.001
	(0.000)	(0.000)	(0.000)	(0.001)
School FE		Yes	Yes	Yes
Controls	Yes	Yes	Yes	Yes

Direct treatment effect (adjusted)	−0.003	−0.015	−0.016	−0.013
P-Value	0.929	0.602	0.566	0.641
Total treatment effect (adjusted)	0.360	−0.503	−0.495	−0.284
P-Value	0.282	0.009	0.031	0.018

Observations	3,946	3,946	3,946	3,944
R${}^{2}$	0.174	0.216	0.218	0.216

Notes: Robust standard errors in parentheses.

Controls include student age, sex, grade, and baseline test scores.

The direct treatment effect is the effect of treatment plus the interaction of treatment with the share of peers that are treated, and the interaction of treatment with peer group size, whilst adjusting for peer effects. The total treatment effect is the direct treatment effect, plus the effect of spillovers independent of individual treatment.

In column 1, treatment is defined as the share treated peers within their school.

In column 2, treatment is the share of treated peers within their half of their school (upper or lower).

In column 3, treatment is the share of treated peers in their grade or the two adjacent grades.

In column 4, treatment is the share of treated peers in their grade alone.

${}^{*}$p<0.10, ${}^{**}$p<0.05, ${}^{***}$p<0.01.

**Table F.2:** Heterogeneous effects by share of treated peers (Language) .

	(1)	(2)	(3)	(4)
	School	Upper/ Lower	Adj grades	Grade
Treatment	−0.432	0.303	0.188	0.182
	(0.559)	(0.306)	(0.324)	(0.192)
Share of treated peers	0.763	−0.571	−0.623	−0.284
	(0.767)	(0.394)	(0.492)	(0.241)
Treatment × Share	0.870	−0.623	−0.450	−0.394
	(1.064)	(0.571)	(0.607)	(0.349)
Peer group size	0.001${}^{***}$	−0.001	0.000	−0.000
	(0.000)	(0.001)	(0.001)	(0.001)
Treatment × Peer group size	−0.000	−0.000	0.000	−0.000
	(0.000)	(0.000)	(0.000)	(0.001)
School FE		Yes	Yes	Yes
Controls	Yes	Yes	Yes	Yes

Direct treatment effect (adjusted)	−0.007	−0.017	−0.018	−0.017
P-Value	0.815	0.541	0.526	0.554
Total treatment effect (adjusted)	0.375	−0.303	−0.330	−0.159
P-Value	0.333	0.133	0.190	0.202

Observations	3,946	3,946	3,946	3,944
R${}^{2}$	0.133	0.211	0.211	0.212

Notes: Robust standard errors in parentheses.

Controls include student age, sex, grade, and baseline test scores.

The direct treatment effect is the effect of treatment plus the interaction of treatment with the share of peers that are treated, and the interaction of treatment with peer group size, whilst adjusting for peer effects. The total treatment effect is the direct treatment effect, plus the effect of spillovers independent of individual treatment.

In column 1, treatment is defined as the share treated peers within their school.

In column 2, treatment is the share of treated peers within their half of their school (upper or lower).

In column 3, treatment is the share of treated peers in their grade or the two adjacent grades.

In column 4, treatment is the share of treated peers in their grade alone.

${}^{*}$p<0.10, ${}^{**}$p<0.05, ${}^{***}$p<0.01.

**Table F.3:** Heterogeneous effects by number of treated peers (Maths) .

	(1)	(2)	(3)	(4)
	School	Upper/ Lower	Adj grades	Grade
Treatment	−0.058	−0.055	−0.039	−0.049
	(0.068)	(0.061)	(0.058)	(0.059)
Number of treated peers	0.003	−0.012${}^{**}$	−0.007	−0.018${}^{***}$
	(0.003)	(0.005)	(0.005)	(0.007)
Treatment × Peers	0.005	0.003	0.002	0.006
	(0.004)	(0.005)	(0.006)	(0.008)
Peer group size	−0.001	0.006${}^{***}$	0.006${}^{**}$	0.012${}^{***}$
	(0.001)	(0.002)	(0.002)	(0.004)
Treatment × Peer group size	−0.002	−0.001	−0.001	−0.002
	(0.002)	(0.002)	(0.003)	(0.004)
School FE		Yes	Yes	Yes
Controls	Yes	Yes	Yes	Yes

Direct treatment effect (adjusted)	−0.740	−0.229	−0.170	−0.168
P-Value	0.213	0.561	0.656	0.445
Total treatment effect (adjusted)	0.440	−0.900	−0.492	−0.496
P-Value	0.306	0.011	0.156	0.006

Observations	3,946	3,946	3,946	3,946
R${}^{2}$	0.174	0.215	0.217	0.217

Notes: Robust standard errors in parentheses.

Controls include student age, sex, grade, and baseline test scores.

The direct treatment effect is the effect of treatment plus the interaction of treatment with the share of peers that are treated, and the interaction of treatment with peer group size, whilst adjusting for peer effects. The total treatment effect is the direct treatment effect, plus the effect of spillovers independent of individual treatment.

In column 1, treatment is defined as the share treated peers within their school.

In column 2, treatment is the share of treated peers within their half of their school (upper or lower).

In column 3, treatment is the share of treated peers in their grade or the two adjacent grades.

In column 4, treatment is the share of treated peers in their grade alone.

${}^{*}$p<0.10, ${}^{**}$p<0.05, ${}^{***}$p<0.01.

**Table F.4:** Heterogeneous effects by number of treated peers (Language) .

	(1)	(2)	(3)	(4)
	School	Upper/ Lower	Adj grades	Grade
Treatment	0.024	0.022	−0.010	−0.004
	(0.073)	(0.064)	(0.061)	(0.061)
Number of treated peers	0.008${}^{***}$	−0.004	−0.002	−0.009
	(0.003)	(0.005)	(0.005)	(0.007)
Treatment × Peers	−0.002	−0.008	−0.007	−0.005
	(0.004)	(0.005)	(0.005)	(0.009)
Peer group size	−0.003${}^{**}$	0.001	0.001	0.004
	(0.001)	(0.002)	(0.002)	(0.004)
Treatment × Peer group size	0.001	0.004	0.003	0.002
	(0.002)	(0.002)	(0.003)	(0.004)
School FE		Yes	Yes	Yes
Controls	Yes	Yes	Yes	Yes

Direct treatment effect (adjusted)	0.248	0.568	0.431	0.120
P-Value	0.686	0.148	0.248	0.594
Total treatment effect (adjusted)	1.238	−0.311	−0.145	−0.267
P-Value	0.006	0.395	0.678	0.146

Observations	3,946	3,946	3,946	3,946
R${}^{2}$	0.134	0.211	0.211	0.211

Notes: Robust standard errors in parentheses.

Controls include student age, sex, grade, and baseline test scores.

The direct treatment effect is the effect of treatment plus the interaction of treatment with the share of peers that are treated, and the interaction of treatment with peer group size, whilst adjusting for peer effects. The total treatment effect is the direct treatment effect, plus the effect of spillovers independent of individual treatment.

In column 1, treatment is defined as the share treated peers within their school.

In column 2, treatment is the share of treated peers within their half of their school (upper or lower).

In column 3, treatment is the share of treated peers in their grade or the two adjacent grades.

In column 4, treatment is the share of treated peers in their grade alone.

${}^{*}$p<0.10, ${}^{**}$p<0.05, ${}^{***}$p<0.01.

**Table F.5:** 2020 school exam pass rates .

	(1)	(2)	(3)
Study Schools	−0.097	−0.054	−0.036
	(0.074)	(0.054)	(0.057)
2019 Pass rate		0.393${}^{***}$	0.381${}^{***}$
		(0.044)	(0.047)
Total students enrolled			−0.000${}^{**}$
			(0.000)
Chiefdom FE			Yes

Observations	373	373	373
R${}^{2}$	0.007	0.268	0.319

Notes: Robust standard errors in parentheses.

This table regresses 2020 National Primary School Examination (NPSE) pass rates on whether the school was included in our study.

${}^{*}$p<0.10, ${}^{**}$p<0.05, ${}^{***}$p<0.01.

## Data Availability

The replication data and do-files for this article are available in the Harvard Dataverse ( https://doi.org/10.7910/DVN/OPQ37C).

## Appendix A Additional figures and tables

See Fig. A.1 and Table A.1, Table A.2, Table A.3, Table A.4, Table A.5, Table A.6, Table A.7, Table A.8, Table A.9.

## Appendix B Individual mathematics and language question items

**Mathematics items**

1. Please could you count from 1 to 10 [Correct/incorrect/Didn’t attempt/refused]
2. What number is bigger, 5 or 2? Enter number as given by the child.
3. What is 4+7? Enter number as given by the child.
4. What is 12 + 5? Enter number as given by the child.
5. What is 24 + 59? Enter number as given by the child.
6. What is 19 − 5? Enter number as given by the child.
7. What is 85 − 13? Enter number as given by the child.
8. “Suppose you have 25 bananas, and I take away 17. What do you have?” Enter number as given by the child.
9. “An onion costs 1000 Leones each. I have 5000 Leones. How many onions can I buy?” Enter number as given by the child.
10. What is 7 × 4? Enter number as given by the child.
11. What is 2 × 13? Enter number as given by the child.
12. What is 9 ÷ 3? Enter number as given by the child.

**Language items**

1. Now let’s try a word game. Imagine you are going to the market. Tell me some things that you can buy from the market. Try to name as many things as you can think of and I will keep count. Keep count of the number of items the child names, up to a maximum of 10 items. If the child pauses for 5 seconds or more, PROMPT ONCE by saying, “Can you think of any others?” When the child cannot think of more items, move on to the next question. Enter the number of items the child was able to name into answer box.
2. Now, I want to know what fruits and vegetables you are familiar with. Tell me the names of fruits and vegetables that you know. Try to name as many fruits and vegetables as you can think of and I will keep count again. When the child pauses for 5 seconds or more, PROMPT ONCE by saying “Can you think of any others?” Enter the number of fruits and vegetables the child was able to name into the answer box.
3. We’re going to try and spell out some words now. Please can you spell the word “cat” for me? Enter word as spelt by the child
4. Please can you spell the word “walk” for me. Enter word as spelt by the child
5. And finally, please can you spell the word “pencil” for me. Enter word as spelt by the child

Now I’m going to tell you a short story and will ask you some questions afterwards. Every day Hassan walks to school with his friend Mariama. On their way to school, the children like to have a race to see who runs the fastest. It is Mariama!

6. Who does Hassan like to walk to school with? Answer = Mariama. [Correct, Incorrect, No Response]
7. What do they do on their way? Answer = they race/run. [Correct, Incorrect, No response]
8. Who runs faster? Answer = Mariama. [Correct, Incorrect, No Response]

Now I am going to tell you another interesting story. This one is a little bit longer. After I have told you the story, I will ask you some questions. Listen carefully, okay? Read out the story slowly, clearly and fluently.

The Mouse and the Cat

Once upon a time there was a fat cat. He always wore a red hat. Once when he was sleeping, a small mouse came silently and stole the cat’s hat. The cat woke up to see his hat gone, got very angry and started chasing the mouse. After a while, the mouse was trapped under a table and could not find any way to escape. So the mouse said to the cat, “Please don’t eat me, cat. If you spare my life I will return your hat”. So, after getting back his hat the cat said, “Please don’t touch my hat again” and he went back to sleep in a happy mood.

Now I am going to ask you some questions about the story.

9. Who stole the cat’s hat? Answer = the mouse. [A Correct/B Incorrect/C Refused/skipped]
10. What colour was the hat? Answer = red. [A Correct/B Incorrect/C Refused/skipped]
11. Why did the cat chase the mouse? Answer = because his hat was gone/missing. [A Correct/B Incorrect/C Refused/skipped]
12. 12. Where did the mouse get trapped? Answer = under a table. [A Correct/B Incorrect/C Refused/skipped]

## Appendix C Example tuition call scripts

*Reviewing content from Week 1 of Lower Primary Literacy Radio Lessons.*

During your conversation with the student, ask each question. Pause and wait for the student to answer. Encourage them to try answering. If they are correct, tell them so. If they are incorrect, tell them it was a good try but not quite right. No matter whether the student is correct or not, tell them the correct answer after they try and encourage them again.

*Reviewing content from Week 1 of Lower Primary Maths Radio Lessons.*

## Appendix E Survey mode

Our findings on assessing student learning by phone show that this is feasible, that our phone assessments have good internal reliability (see the discussion in Section 5.4 and Table E.1, Table E.2, Table E.3, Table E.4), and that the mode of assessment has no significant impact on treatment effect estimates (see the discussion in Section 6.3 and results in Table E.5). Our comparison of phone-based assessment and in-person assessment is hindered, however, by low adherence to survey mode random assignment. As a result, we cannot make conclusive statements about the effectiveness of phone-based versus in-person assessments. We see a large negative correlation between being surveyed in-person and test scores, but this is not apparent in the randomised allocation or when using randomised assignment as an instrument for actual survey mode. We also see selection in which households participate in phone-based assessments. Although the phone survey has good internal consistency, we do see differences in item functioning between phone and in-person surveys.

We see substantial mode effects in the OLS specification. Students surveyed in-person scored −0.43 standard deviations worse in mathematics than those surveyed by phone. These differences could though be driven by selection as much as by the survey mode itself. We do not see these mode effects with the randomly assigned intent to be surveyed in-person, but this may be due to low compliance with the randomisation. Of 499 children randomly assigned to be surveyed in-person, 233 were found and surveyed in-person, and 186 were surveyed by phone. An additional 1429 students originally planned to be surveyed by phone (but who were unreachable) were then found and surveyed in-person at their school. Random assignment to in-person interview increased the probability that an individual was actually interviewed in-person by 11 percentage points. Including control variables in the instrumental variable specification changes the sign of the estimated effect of in-person assessment, but these estimates have large standard errors and are mostly not statistically significant (Table E.6).^7^ Qualitative evidence suggests that differences between in-person and phone learning assessment scores may be in part explained by students interviewed by phone being helped by their parents, despite the request of interviewers not to do so (Sam-Kpakra, 2021). *“As for me what I mostly did was when they ask my child and he doesn’t know the answer, I push the phone far away and tell him the answer”.* (Parent, Kenema District)

## Appendix F Potential spillovers

In this section we present evidence on the potential for spillovers between students. We first regress students’ own test scores, Y, on their own treatment status, T, and on the share of their peers who are treated, S. Note that these shares vary randomly, due to random assignment of individual pupils, though variation is somewhat limited: at the grade level, the 10th percentile of S is roughly 42% and the 90th percentile is 61%.

The estimating equation used in Table F.1, Table F.2 is

$$Y_{ig}=\beta_{1}\;T_{ig}+\beta_{2}S_{ig}+\beta_{3}\left(T_{ig}*S_{ig}\right)+\beta_{4}P_{ig}+\beta_{5}\left(T_{ig}*P_{ig}\right)+\epsilon_{ig}$$

for pupil i in peer group g. P is a control for the size of the peer group. We define the “direct” treatment effect as the impact of treatment per se, controlling for the presence of spillovers, i.e.,

$$\partial Y_{ig}/\partial T_{ig}=\beta_{1}+\beta_{3}S_{ig}+\beta_{5}P_{ig},$$

where S and P are evaluated at the sample mean. In contrast, we define the *total* treatment effect as the direct effect of treatment plus spillovers, or

$$\beta_{1}+\beta_{2}S_{ig}+\beta_{3}S_{ig}+\beta_{5}P_{ig}.$$

The estimating equation used in Table F.3, Table F.4 is

$$Y_{ig}=\beta_{1}\;T_{ig}+\beta_{2}C_{ig}+\beta_{3}\left(T_{ig}*C_{ig}\right)+\beta_{4}P_{ig}+\beta_{5}\left(T_{ig}*P_{ig}\right)+\epsilon_{ig}$$

where C is the count, rather than the share, of the number of treated peers.

## Footnotes

2 We discuss potential ethical issues with the design in Appendix D.

3 We had intended to assign a fourth group as a pure control group to receive neither SMS reminders nor tutoring calls, however in practice this group also received SMS reminders and so we include them in the SMS only group.

4 Although 499 is a less than ideal sample size, but we were constrained by budget and what logistics seemed feasible during the uncertainty of the pandemic. Some evidence suggests that the item-response theory (IRT) models we use can be feasibly estimated with samples as small as 150 (Şahin and Anıl, 2017).

6 While the negative spillover effects are not statistically significant, negative spillovers could be explained either by a displacement effect, such that the treatment displaced potential calls from control students by focusing teachers on treatment students, or a demoralisation effect, such that control pupils heard that other pupils were receiving special treatment and subsequently exerted less effort.

## Footnotes

5 We also show the DIF graphically in Fig. E.1, Fig. E.2, and the full distribution of test scores by survey mode and assignment in Fig. E.3.
